# Bis(formato-κ*O*)bis­[1-(pyridin-2-yl)ethanone oxime-κ^2^ *N*,*N*′]nickel(II)

**DOI:** 10.1107/S1600536811050859

**Published:** 2011-12-03

**Authors:** Leilei Li, Xiumin Qiu, Dacheng Li

**Affiliations:** a College of Chemistry and Chemical Engineering, Liaocheng University, Shandong 252059, People’s Republic of China

## Abstract

In the title compound, [Ni(HCOO)_2_(C_7_H_8_N_2_O)_2_], the Ni atom is six-coordinated by four N atoms from two oxime ligands and by two O atoms from two formate ions in a distorted octa­hedral geometry, with the oxime-N atoms mutually *trans*. The mol­ecular conformation is stabilized by intra­molecular O—H⋯O hydrogen bonds.

## Related literature

For uses of oximes, see: Davidson *et al.* (2007 ▶); Pavlishchuk *et al.* (2003 ▶) and of 2-pyridyl oximes, see: Chaudhuri (2003 ▶); Milios *et al.* (2006 ▶). For a related structure, see: Zuo *et al.* (2007 ▶). 
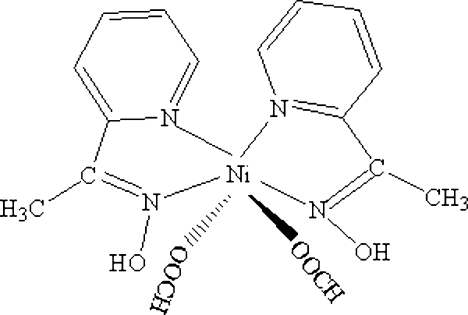


## Experimental

### 

#### Crystal data

[Ni(CHO_2_)_2_(C_7_H_8_N_2_O)_2_]
*M* *_r_* = 421.05
Monoclinic, 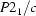

*a* = 10.5000 (12) Å
*b* = 14.6109 (16) Å
*c* = 15.7391 (17) Å
β = 131.850 (2)°
*V* = 1798.6 (3) Å^3^
*Z* = 4
Mo *K*α radiation
μ = 1.12 mm^−1^
*T* = 298 K
0.20 × 0.17 × 0.11 mm

#### Data collection

Bruker SMART CCD area-detector diffractometer
Absorption correction: multi-scan (*SADABS*; Sheldrick, 1996 ▶) *T*_min_ = 0.807, *T*_max_ = 0.887
9294 measured reflections
3171 independent reflections
2209 reflections with *I* > 2σ(*I*)
*R*_int_ = 0.077

#### Refinement

*R*[*F* ^2^ > 2σ(*F* ^2^)] = 0.049
*wR*(*F* ^2^) = 0.137
*S* = 1.00
3171 reflections
244 parameters
H-atom parameters constrained
Δρ_max_ = 0.98 e Å^−3^
Δρ_min_ = −0.38 e Å^−3^

### 

Data collection: *SMART* (Bruker, 2007 ▶); cell refinement: *SAINT* (Bruker, 2007 ▶); data reduction: *SAINT*; program(s) used to solve structure: *SHELXS97* (Sheldrick, 2008 ▶); program(s) used to refine structure: *SHELXL97* (Sheldrick, 2008 ▶); molecular graphics: *SHELXTL* (Sheldrick, 2008 ▶); software used to prepare material for publication: *SHELXTL*.

## Supplementary Material

Crystal structure: contains datablock(s) I, global. DOI: 10.1107/S1600536811050859/vm2139sup1.cif

Structure factors: contains datablock(s) I. DOI: 10.1107/S1600536811050859/vm2139Isup2.hkl

Additional supplementary materials:  crystallographic information; 3D view; checkCIF report

## Figures and Tables

**Table 1:** Hydrogen-bond geometry (Å, °)

*D*—H⋯*A*	*D*—H	H⋯*A*	*D*⋯*A*	*D*—H⋯*A*
O1—H1⋯O4	0.82	1.69	2.508 (4)	177
O2—H2⋯O6	0.82	1.67	2.482 (4)	169

**Fig. 1.**
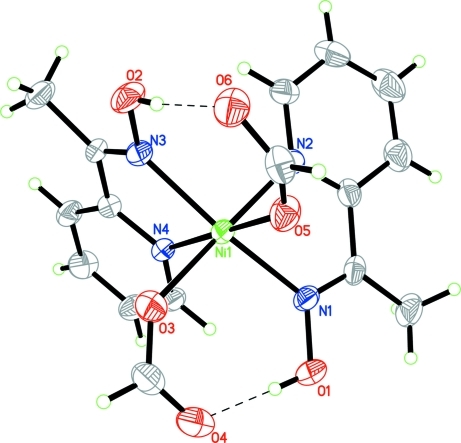
The molecular structure of the title compound, with atom labels and 30% probability displacement ellipsoids.

## supplementary crystallographic information

### Comment

Recently, there is a intense interest in the coordination chemistry of oximes (Davidson *et al.*, 2007; Pavlishchuk *et al.*, 2003). 2-Pyridyl oximes which are versatile ligands for a variety of research objectives are popular ligands in coordination chemistry (Chaudhuri, 2003; Milios *et al.*, 2006). We report here the synthesis and structure of the title compound.

The complex (Fig. 1) crystallizes in monoclinic space group *P*2_1_/*c*. The central Ni atom is six coordinated by four N atoms from the oxime ligands and two O atoms from the formate ions in a distorted octahedral geometry. The N1 and N3 atoms of methyl 2-pyridylketone oxime occupy the axial sites. The N2, N4 and O3, O5 are in the equatorial plane. The six coordinated molecule is the *cis*-*cis*-*trans* isomer considering the positions of the coordinated formyl groups, pyridyl and oxime nitrogen atoms, respectively (Zuo *et al.*, 2007). The Ni–N bond distances in the compound are in the range of 2.075 (3)–2.107 (3) Å which is longer than the Ni–O distances (2.049 (3)–2.070 (3) Å). The molecular conformation is stabilized by intramolecular O—H···O hydrogen bonds (Table 1).

### Experimental

A solution of NiSO_4_.6H_2_O (0.131 g, 0.5 mmol) and methyl 2-pyridyl ketone oxime (0.068 g, 0.5 mmol) in MeOH was treated with equivalent amounts of HCOONa. After stirring for 6 h, a green solution was obtained. After filtration, green block crystals suitable for single-crystal X-ray diffraction were obtained after two weeks by evaporating the resulting filtrate in air. Yield: 47% (based on Ni).

### Refinement

All H atoms were placed geometrically and treated as riding on their parent atoms with O—H = 0.82 Å [*U*_iso_(H) = 1.5*U*_eq_(O)], C—H = 0.97 (methyl) Å [*U*_iso_(H) = 1.5*U*_eq_(C)], and C—H = 0.93 (aromatic and formic) Å [*U*_iso_(H) = 1.2*U*_eq_(C)].

### Crystal data

**Table:**

[Ni(CHO_2_)_2_(C_7_H_8_N_2_O)_2_]	*F*(000) = 872
*M**_r_* = 421.05	*D*_x_ = 1.555 Mg m^−^^3^
Monoclinic, *P*2_1_/*c*	Mo *K*α radiation, λ = 0.71073 Å
Hall symbol: -P 2ybc	Cell parameters from 3030 reflections
*a* = 10.5000 (12) Å	θ = 2.4–25.3°
*b* = 14.6109 (16) Å	µ = 1.12 mm^−^^1^
*c* = 15.7391 (17) Å	*T* = 298 K
β = 131.850 (2)°	Block, green
*V* = 1798.6 (3) Å^3^	0.20 × 0.17 × 0.11 mm
*Z* = 4	

### Data collection

**Table:**

Bruker SMART CCD area-detector diffractometer	3171 independent reflections
Radiation source: fine-focus sealed tube	2209 reflections with *I* > 2σ(*I*)
graphite	*R*_int_ = 0.077
phi and ω scans	θ_max_ = 25.0°, θ_min_ = 2.2°
Absorption correction: multi-scan (*SADABS*; Sheldrick, 1996)	*h* = −12→11
*T*_min_ = 0.807, *T*_max_ = 0.887	*k* = −16→17
9294 measured reflections	*l* = −14→18

### Refinement

**Table:**

Refinement on *F*^2^	Primary atom site location: structure-invariant direct methods
Least-squares matrix: full	Secondary atom site location: difference Fourier map
*R*[*F*^2^ > 2σ(*F*^2^)] = 0.049	Hydrogen site location: inferred from neighbouring sites
*wR*(*F*^2^) = 0.137	H-atom parameters constrained
*S* = 1.00	*w* = 1/[σ^2^(*F*_o_^2^) + (0.077*P*)^2^] where *P* = (*F*_o_^2^ + 2*F*_c_^2^)/3
3171 reflections	(Δ/σ)_max_ < 0.001
244 parameters	Δρ_max_ = 0.98 e Å^−^^3^
0 restraints	Δρ_min_ = −0.38 e Å^−^^3^

### Special details

**Table:**

Geometry. All e.s.d.'s (except the e.s.d. in the dihedral angle between two l.s. planes) are estimated using the full covariance matrix. The cell e.s.d.'s are taken into account individually in the estimation of e.s.d.'s in distances, angles and torsion angles; correlations between e.s.d.'s in cell parameters are only used when they are defined by crystal symmetry. An approximate (isotropic) treatment of cell e.s.d.'s is used for estimating e.s.d.'s involving l.s. planes.
Refinement. Refinement of *F*^2^ against ALL reflections. The weighted *R*-factor *wR* and goodness of fit *S* are based on *F*^2^, conventional *R*-factors *R* are based on *F*, with *F* set to zero for negative *F*^2^. The threshold expression of *F*^2^ > σ(*F*^2^) is used only for calculating *R*-factors(gt) *etc*. and is not relevant to the choice of reflections for refinement. *R*-factors based on *F*^2^ are statistically about twice as large as those based on *F*, and *R*- factors based on ALL data will be even larger.

### Fractional atomic coordinates and isotropic or equivalent isotropic displacement parameters (Å^2^)

**Table:**

	*x*	*y*	*z*	*U*_iso_*/*U*_eq_	
Ni1	0.79945 (5)	0.29154 (3)	0.43579 (3)	0.0370 (2)	
N1	0.9617 (4)	0.2714 (2)	0.6114 (2)	0.0438 (8)	
N2	0.9641 (4)	0.1861 (2)	0.4685 (2)	0.0417 (7)	
N3	0.6356 (4)	0.2740 (2)	0.2604 (2)	0.0425 (7)	
N4	0.6175 (3)	0.19950 (19)	0.4010 (2)	0.0389 (7)	
O1	0.9530 (4)	0.3159 (2)	0.6839 (2)	0.0625 (8)	
H1	0.8715	0.3512	0.6473	0.094*	
O2	0.6505 (4)	0.3135 (2)	0.1895 (2)	0.0600 (8)	
H2	0.7258	0.3525	0.2244	0.090*	
O3	0.6415 (3)	0.39657 (19)	0.4059 (2)	0.0594 (7)	
O4	0.6976 (4)	0.4197 (3)	0.5686 (3)	0.0824 (11)	
O5	0.9714 (3)	0.38535 (19)	0.4670 (2)	0.0565 (7)	
O6	0.9048 (4)	0.4151 (2)	0.3023 (2)	0.0647 (8)	
C1	1.1889 (5)	0.1813 (3)	0.7810 (3)	0.0666 (13)	
H1A	1.1802	0.2260	0.8217	0.100*	
H1B	1.3051	0.1780	0.8133	0.100*	
H1C	1.1540	0.1226	0.7866	0.100*	
C2	1.0756 (4)	0.2085 (3)	0.6584 (3)	0.0453 (9)	
C3	1.0842 (4)	0.1614 (3)	0.5792 (3)	0.0454 (9)	
C4	1.2087 (5)	0.0964 (3)	0.6154 (4)	0.0640 (12)	
H4	1.2920	0.0811	0.6921	0.077*	
C5	1.2066 (6)	0.0551 (3)	0.5356 (4)	0.0763 (14)	
H5	1.2880	0.0113	0.5578	0.092*	
C6	1.0840 (6)	0.0793 (3)	0.4241 (4)	0.0692 (13)	
H6	1.0803	0.0518	0.3692	0.083*	
C7	0.9647 (5)	0.1451 (3)	0.3928 (3)	0.0527 (10)	
H7	0.8819	0.1614	0.3164	0.063*	
C8	0.3945 (5)	0.1940 (3)	0.0882 (3)	0.0625 (12)	
H8A	0.3672	0.2490	0.0455	0.094*	
H8B	0.2915	0.1674	0.0643	0.094*	
H8C	0.4493	0.1514	0.0750	0.094*	
C9	0.5122 (4)	0.2164 (2)	0.2125 (3)	0.0399 (8)	
C10	0.4964 (4)	0.1737 (3)	0.2905 (3)	0.0398 (8)	
C11	0.3649 (5)	0.1158 (3)	0.2541 (3)	0.0508 (10)	
H11	0.2837	0.0982	0.1780	0.061*	
C12	0.3560 (5)	0.0843 (3)	0.3330 (4)	0.0570 (11)	
H12	0.2686	0.0449	0.3105	0.068*	
C13	0.4765 (5)	0.1115 (3)	0.4443 (3)	0.0535 (10)	
H13	0.4710	0.0921	0.4981	0.064*	
C14	0.6058 (4)	0.1680 (3)	0.4749 (3)	0.0428 (9)	
H14	0.6892	0.1852	0.5510	0.051*	
C15	0.6164 (5)	0.4323 (3)	0.4662 (4)	0.0604 (11)	
H15	0.5257	0.4733	0.4293	0.072*	
C16	0.9880 (5)	0.4261 (3)	0.4058 (4)	0.0535 (10)	
H16	1.0734	0.4703	0.4419	0.064*	

### Atomic displacement parameters (Å^2^)

**Table:**

	*U*^11^	*U*^22^	*U*^33^	*U*^12^	*U*^13^	*U*^23^
Ni1	0.0332 (3)	0.0470 (3)	0.0287 (3)	−0.0006 (2)	0.0199 (2)	−0.00075 (19)
N1	0.0397 (17)	0.061 (2)	0.0314 (16)	−0.0092 (15)	0.0238 (15)	−0.0067 (14)
N2	0.0377 (16)	0.0543 (19)	0.0363 (17)	0.0045 (13)	0.0260 (15)	0.0063 (13)
N3	0.0422 (17)	0.055 (2)	0.0331 (16)	0.0040 (15)	0.0263 (15)	0.0067 (14)
N4	0.0350 (16)	0.0485 (18)	0.0303 (15)	−0.0008 (13)	0.0206 (14)	−0.0040 (12)
O1	0.0598 (17)	0.091 (2)	0.0364 (15)	0.0007 (15)	0.0322 (14)	−0.0092 (14)
O2	0.0632 (18)	0.082 (2)	0.0373 (15)	−0.0115 (15)	0.0347 (14)	0.0029 (13)
O3	0.0552 (17)	0.0595 (18)	0.0588 (18)	0.0075 (13)	0.0361 (15)	−0.0041 (14)
O4	0.083 (2)	0.104 (3)	0.073 (2)	0.022 (2)	0.058 (2)	−0.0027 (19)
O5	0.0495 (16)	0.0650 (19)	0.0470 (16)	−0.0112 (13)	0.0289 (14)	0.0010 (13)
O6	0.0609 (18)	0.074 (2)	0.0571 (19)	−0.0065 (16)	0.0386 (16)	0.0083 (16)
C1	0.051 (3)	0.095 (3)	0.037 (2)	0.004 (2)	0.022 (2)	0.012 (2)
C2	0.0305 (19)	0.062 (3)	0.033 (2)	−0.0076 (18)	0.0172 (17)	0.0042 (17)
C3	0.035 (2)	0.055 (2)	0.044 (2)	0.0015 (17)	0.0258 (18)	0.0078 (18)
C4	0.052 (3)	0.077 (3)	0.058 (3)	0.014 (2)	0.034 (2)	0.020 (2)
C5	0.071 (3)	0.078 (3)	0.091 (4)	0.033 (3)	0.059 (3)	0.026 (3)
C6	0.080 (3)	0.074 (3)	0.083 (4)	0.012 (3)	0.066 (3)	0.000 (3)
C7	0.055 (2)	0.063 (3)	0.050 (2)	0.007 (2)	0.039 (2)	0.003 (2)
C8	0.056 (3)	0.087 (3)	0.032 (2)	−0.012 (2)	0.024 (2)	−0.012 (2)
C9	0.0332 (19)	0.052 (2)	0.0245 (17)	0.0053 (17)	0.0151 (16)	−0.0010 (15)
C10	0.0371 (19)	0.045 (2)	0.0356 (19)	0.0024 (16)	0.0237 (17)	−0.0014 (16)
C11	0.045 (2)	0.058 (3)	0.039 (2)	−0.0116 (18)	0.0237 (18)	−0.0149 (18)
C12	0.058 (3)	0.057 (3)	0.064 (3)	−0.019 (2)	0.044 (2)	−0.012 (2)
C13	0.062 (3)	0.057 (3)	0.049 (2)	−0.011 (2)	0.040 (2)	−0.0053 (19)
C14	0.042 (2)	0.054 (2)	0.0329 (19)	−0.0052 (17)	0.0250 (18)	−0.0043 (17)
C15	0.052 (3)	0.057 (3)	0.077 (3)	−0.002 (2)	0.045 (3)	−0.011 (2)
C16	0.042 (2)	0.050 (2)	0.062 (3)	0.0012 (18)	0.032 (2)	0.008 (2)

### Geometric parameters (Å, °)

**Table:**

Ni1—O5	2.049 (3)	C2—C3	1.478 (5)
Ni1—O3	2.070 (3)	C3—C4	1.394 (5)
Ni1—N3	2.075 (3)	C4—C5	1.379 (7)
Ni1—N1	2.085 (3)	C4—H4	0.9300
Ni1—N4	2.089 (3)	C5—C6	1.359 (6)
Ni1—N2	2.107 (3)	C5—H5	0.9300
N1—C2	1.281 (5)	C6—C7	1.382 (5)
N1—O1	1.368 (4)	C6—H6	0.9300
N2—C7	1.337 (5)	C7—H7	0.9300
N2—C3	1.351 (4)	C8—C9	1.494 (5)
N3—C9	1.285 (4)	C8—H8A	0.9600
N3—O2	1.356 (4)	C8—H8B	0.9600
N4—C14	1.330 (4)	C8—H8C	0.9600
N4—C10	1.354 (4)	C9—C10	1.481 (5)
O1—H1	0.8200	C10—C11	1.377 (5)
O2—H2	0.8200	C11—C12	1.386 (5)
O3—C15	1.256 (5)	C11—H11	0.9300
O4—C15	1.235 (5)	C12—C13	1.367 (5)
O5—C16	1.239 (5)	C12—H12	0.9300
O6—C16	1.243 (5)	C13—C14	1.373 (5)
C1—C2	1.493 (5)	C13—H13	0.9300
C1—H1A	0.9600	C14—H14	0.9300
C1—H1B	0.9600	C15—H15	0.9300
C1—H1C	0.9600	C16—H16	0.9300
			
O5—Ni1—O3	90.17 (12)	C5—C4—C3	119.0 (4)
O5—Ni1—N3	102.57 (11)	C5—C4—H4	120.5
O3—Ni1—N3	87.81 (11)	C3—C4—H4	120.5
O5—Ni1—N1	88.09 (11)	C6—C5—C4	119.2 (4)
O3—Ni1—N1	103.18 (12)	C6—C5—H5	120.4
N3—Ni1—N1	164.78 (12)	C4—C5—H5	120.4
O5—Ni1—N4	177.98 (11)	C5—C6—C7	119.5 (4)
O3—Ni1—N4	87.92 (11)	C5—C6—H6	120.2
N3—Ni1—N4	76.72 (11)	C7—C6—H6	120.2
N1—Ni1—N4	92.98 (11)	N2—C7—C6	122.4 (4)
O5—Ni1—N2	88.99 (12)	N2—C7—H7	118.8
O3—Ni1—N2	178.97 (12)	C6—C7—H7	118.8
N3—Ni1—N2	92.95 (11)	C9—C8—H8A	109.5
N1—Ni1—N2	76.20 (11)	C9—C8—H8B	109.5
N4—Ni1—N2	92.93 (12)	H8A—C8—H8B	109.5
C2—N1—O1	114.6 (3)	C9—C8—H8C	109.5
C2—N1—Ni1	119.3 (3)	H8A—C8—H8C	109.5
O1—N1—Ni1	126.0 (2)	H8B—C8—H8C	109.5
C7—N2—C3	118.3 (3)	N3—C9—C10	114.5 (3)
C7—N2—Ni1	126.9 (3)	N3—C9—C8	122.8 (3)
C3—N2—Ni1	114.7 (2)	C10—C9—C8	122.7 (3)
C9—N3—O2	114.8 (3)	N4—C10—C11	121.8 (3)
C9—N3—Ni1	118.6 (2)	N4—C10—C9	114.8 (3)
O2—N3—Ni1	126.4 (2)	C11—C10—C9	123.3 (3)
C14—N4—C10	118.2 (3)	C10—C11—C12	118.7 (3)
C14—N4—Ni1	126.6 (2)	C10—C11—H11	120.6
C10—N4—Ni1	115.2 (2)	C12—C11—H11	120.6
N1—O1—H1	109.5	C13—C12—C11	119.6 (4)
N3—O2—H2	109.5	C13—C12—H12	120.2
C15—O3—Ni1	132.6 (3)	C11—C12—H12	120.2
C16—O5—Ni1	133.9 (3)	C12—C13—C14	118.6 (4)
C2—C1—H1A	109.5	C12—C13—H13	120.7
C2—C1—H1B	109.5	C14—C13—H13	120.7
H1A—C1—H1B	109.5	N4—C14—C13	123.2 (3)
C2—C1—H1C	109.5	N4—C14—H14	118.4
H1A—C1—H1C	109.5	C13—C14—H14	118.4
H1B—C1—H1C	109.5	O4—C15—O3	127.8 (4)
N1—C2—C3	113.9 (3)	O4—C15—H15	116.1
N1—C2—C1	124.6 (4)	O3—C15—H15	116.1
C3—C2—C1	121.4 (4)	O5—C16—O6	128.1 (4)
N2—C3—C4	121.5 (4)	O5—C16—H16	115.9
N2—C3—C2	115.8 (3)	O6—C16—H16	115.9
C4—C3—C2	122.7 (4)		
			
O5—Ni1—N1—C2	−91.6 (3)	O3—Ni1—O5—C16	−87.8 (4)
O3—Ni1—N1—C2	178.7 (3)	N3—Ni1—O5—C16	0.0 (4)
N3—Ni1—N1—C2	43.4 (6)	N1—Ni1—O5—C16	169.0 (4)
N4—Ni1—N1—C2	90.2 (3)	N4—Ni1—O5—C16	−69 (3)
N2—Ni1—N1—C2	−2.1 (3)	N2—Ni1—O5—C16	92.8 (4)
O5—Ni1—N1—O1	92.8 (3)	O1—N1—C2—C3	−179.6 (3)
O3—Ni1—N1—O1	3.1 (3)	Ni1—N1—C2—C3	4.3 (4)
N3—Ni1—N1—O1	−132.2 (4)	O1—N1—C2—C1	1.9 (5)
N4—Ni1—N1—O1	−85.4 (3)	Ni1—N1—C2—C1	−174.1 (3)
N2—Ni1—N1—O1	−177.7 (3)	C7—N2—C3—C4	1.3 (5)
O5—Ni1—N2—C7	−89.8 (3)	Ni1—N2—C3—C4	−176.2 (3)
O3—Ni1—N2—C7	−125 (6)	C7—N2—C3—C2	−179.4 (3)
N3—Ni1—N2—C7	12.8 (3)	Ni1—N2—C3—C2	3.0 (4)
N1—Ni1—N2—C7	−178.0 (3)	N1—C2—C3—N2	−4.8 (5)
N4—Ni1—N2—C7	89.6 (3)	C1—C2—C3—N2	173.7 (3)
O5—Ni1—N2—C3	87.6 (3)	N1—C2—C3—C4	174.5 (4)
O3—Ni1—N2—C3	53 (6)	C1—C2—C3—C4	−7.0 (6)
N3—Ni1—N2—C3	−169.9 (2)	N2—C3—C4—C5	−1.3 (6)
N1—Ni1—N2—C3	−0.7 (2)	C2—C3—C4—C5	179.4 (4)
N4—Ni1—N2—C3	−93.0 (3)	C3—C4—C5—C6	0.4 (7)
O5—Ni1—N3—C9	178.4 (3)	C4—C5—C6—C7	0.5 (7)
O3—Ni1—N3—C9	−91.9 (3)	C3—N2—C7—C6	−0.4 (6)
N1—Ni1—N3—C9	44.9 (5)	Ni1—N2—C7—C6	176.8 (3)
N4—Ni1—N3—C9	−3.5 (3)	C5—C6—C7—N2	−0.5 (7)
N2—Ni1—N3—C9	88.8 (3)	O2—N3—C9—C10	−179.8 (3)
O5—Ni1—N3—O2	2.9 (3)	Ni1—N3—C9—C10	4.1 (4)
O3—Ni1—N3—O2	92.5 (3)	O2—N3—C9—C8	0.3 (5)
N1—Ni1—N3—O2	−130.7 (4)	Ni1—N3—C9—C8	−175.7 (3)
N4—Ni1—N3—O2	−179.1 (3)	C14—N4—C10—C11	−0.8 (5)
N2—Ni1—N3—O2	−86.8 (3)	Ni1—N4—C10—C11	−177.8 (3)
O5—Ni1—N4—C14	−105 (3)	C14—N4—C10—C9	176.2 (3)
O3—Ni1—N4—C14	−86.4 (3)	Ni1—N4—C10—C9	−0.8 (4)
N3—Ni1—N4—C14	−174.6 (3)	N3—C9—C10—N4	−2.1 (5)
N1—Ni1—N4—C14	16.7 (3)	C8—C9—C10—N4	177.7 (3)
N2—Ni1—N4—C14	93.1 (3)	N3—C9—C10—C11	174.8 (3)
O5—Ni1—N4—C10	72 (3)	C8—C9—C10—C11	−5.3 (6)
O3—Ni1—N4—C10	90.4 (2)	N4—C10—C11—C12	0.9 (6)
N3—Ni1—N4—C10	2.2 (2)	C9—C10—C11—C12	−175.8 (4)
N1—Ni1—N4—C10	−166.5 (2)	C10—C11—C12—C13	0.3 (6)
N2—Ni1—N4—C10	−90.2 (2)	C11—C12—C13—C14	−1.4 (6)
O5—Ni1—O3—C15	−100.5 (4)	C10—N4—C14—C13	−0.5 (5)
N3—Ni1—O3—C15	156.9 (4)	Ni1—N4—C14—C13	176.2 (3)
N1—Ni1—O3—C15	−12.4 (4)	C12—C13—C14—N4	1.6 (6)
N4—Ni1—O3—C15	80.1 (4)	Ni1—O3—C15—O4	9.0 (7)
N2—Ni1—O3—C15	−66 (6)	Ni1—O5—C16—O6	−5.8 (7)

### Hydrogen-bond geometry (Å, °)

**Table:**

*D*—H···*A*	*D*—H	H···*A*	*D*···*A*	*D*—H···*A*
O1—H1···O4	0.82	1.69	2.508 (4)	177.
O2—H2···O6	0.82	1.67	2.482 (4)	169.
